# Microbial systems for azo dye biodegradation: enzymatic mechanisms, microbial consortia, and emerging biotechnological strategies

**DOI:** 10.1186/s12934-026-03017-7

**Published:** 2026-05-23

**Authors:** Manar K. Abd Elnabi, Marwa Eltarahony, Abdullah M. El-Badry, Amr Nassrallah, Yehia A.-G. Mahmoud, Mohamed A. Ghazy, Ahmed Kamal, Sameh S. Ali

**Affiliations:** 1 Biotechnology Program, Basic and Applied Science Institute, Egypt-Japan University of Science and Technology (E-JUST), New Borg El-Arab, 21934 Alexandria Egypt; 2 Botany and Microbiology Department, Faculty of Science, Tanta University, Tanta, 31527 Egypt; 3 Environmental Biotechnology Department, Genetic Engineering and Biotechnology Research Institute (GEBRI), City of Scientific Research and Technological Applications (SRTA-City), New Borg El-Arab, 21934 Alexandria Egypt; 4 Environment and Bio-Agriculture Department, Faculty of Agriculture, Al-Azhar University, Cairo, Egypt; 5 Biochemistry Department, Faculty of Agriculture, Cairo University, Giza, 12613 Egypt; 6 Biochemistry Department, Faculty of Science, Ain Shams University, Cairo, 11566 Egypt; 7 Biochemistry Department, Faculty of Science, Suez University, P. O. Box 43518, Suez, Egypt

**Keywords:** Azo dye degradation, Microbial cell factories, Bioremediation, Azoreductases, Enzyme engineering, Microbial consortia, Wastewater treatment, Nanobiotechnology

## Abstract

Azo dyes are the most widely used class of synthetic colorants in textile and related industries; however, their discharge into natural ecosystems poses severe environmental and human-health concerns due to their xenobiotic structure, toxicity, and resistance to degradation. Traditional physicochemical remediation strategies are often costly and may result in incomplete mineralization and secondary pollution. Microbial systems provide an ecologically compatible and economically viable solution through efficient enzymatic reduction and subsequent mineralization of azo dyes and their degradation intermediates. This review synthesizes current advances in microbial bioremediation, with particular emphasis on the enzymatic mechanisms and metabolic processes involved in azo dye degradation, including the roles of key enzymes such as azoreductases, laccases, and peroxidases. The synergistic performance of microbial consortia, optimization of environmental and nutritional parameters, and the integration of bioelectrochemical systems are also discussed. Recent innovations—including genetic engineering, advanced immobilized biocatalysts, nanobiotechnology, high-performance bioreactors, and artificial-intelligence-driven process optimization—are evaluated for their potential to enhance biodegradation efficiency and operational stability. Finally, the major challenges and future perspectives for developing robust microbial systems capable of efficient detoxification and mineralization of azo dyes in industrial wastewater are highlighted. Overall, this review emphasizes the potential of microbial systems and emerging biotechnological strategies as sustainable solutions for azo dye remediation and environmentally responsible wastewater management.

## Introduction

The textile industry is one of the largest industrial sectors worldwide, playing a significant role in economic development and employment while simultaneously representing one of the most water-intensive manufacturing activities [1]. Dyeing and finishing processes consume enormous quantities of water—approximately 200–270 tons for each ton of fabric produced—resulting in the discharge of large volumes of dye-laden wastewater into the environment, often without adequate treatment [2, 3]. It is estimated that textile industries alone release nearly 200,000 tons of dye-contaminated effluents annually, making them a major contributor to aquatic pollution [4, 5].

**Fig. 1:**
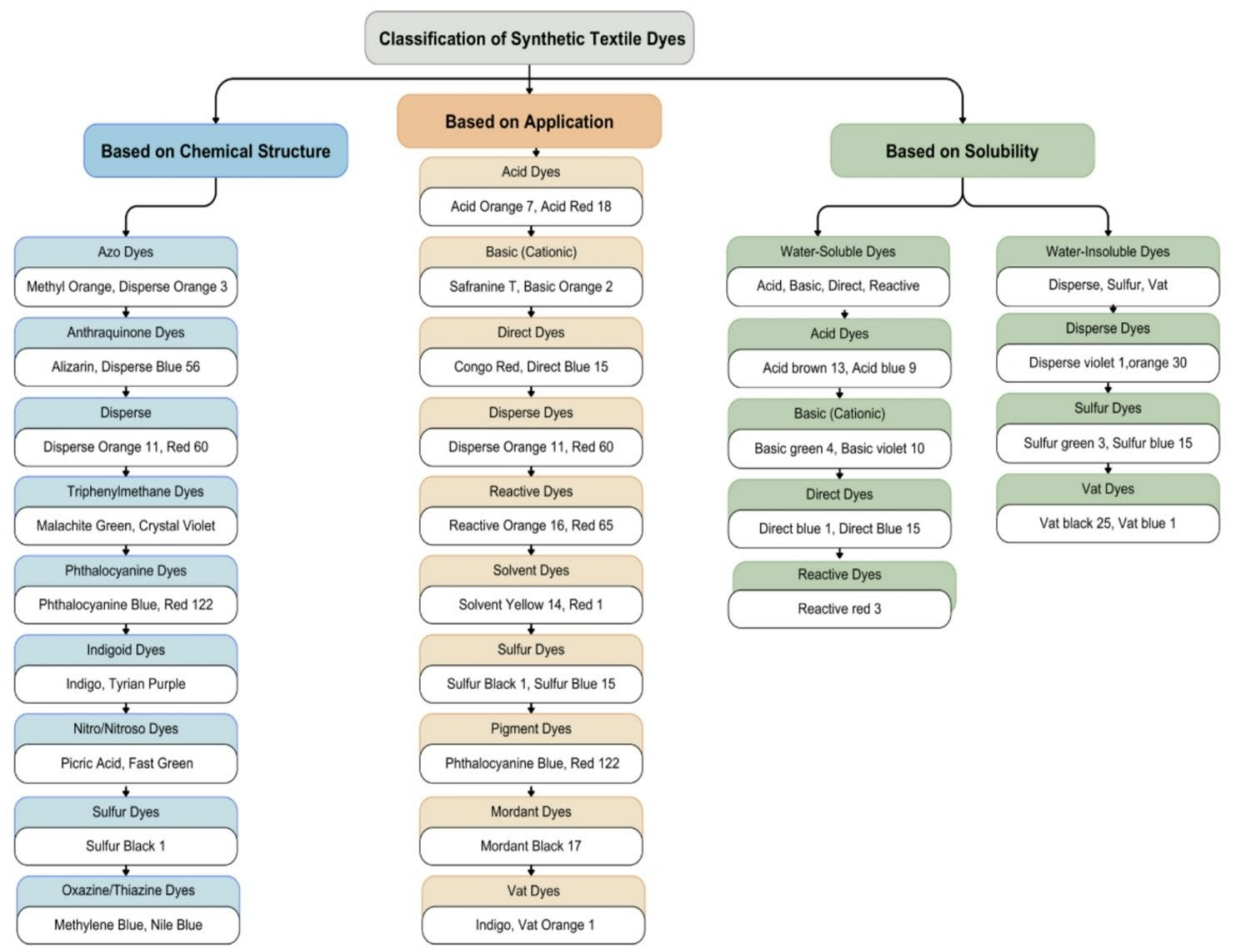
Classification of synthetic textile dyes based on chemical structure, application, and solubility. Dye classes are categorized according to their molecular structure (e.g., azo, anthraquinone, and phthalocyanine dyes), their application in textile processing (e.g., acid, basic, direct, reactive, and vat dyes), and their solubility properties (water-soluble and water-insoluble dyes), along with representative examples of each class

Synthetic dyes, particularly azo dyes, dominate modern industrial applications because of their vivid coloration, low production cost, structural versatility, and strong affinity toward textile fibers [6–9]. The classification of synthetic dyes can be comprehensively described based on their application domains, chemical structure, and solubility properties, as illustrated in Fig. 1. Among these dye classes, azo dyes are the most widely used, accounting for approximately 60–70% of all synthetic dyes employed in textile manufacturing [7, 10]. Structurally, azo dyes contain one or more azo bonds (–N = N–) conjugated with aromatic rings, which confer remarkable chemical stability and resistance to biodegradation [11, 12]. During textile dyeing processes, up to 30% of the applied dyes remain unfixed and are discharged into wastewater streams, where they persist as hazardous xenobiotic pollutants [13, 14].

The release of azo dye–containing effluents poses severe ecological and public-health risks. Even at low concentrations, the intense coloration of these dyes reduces light penetration into water bodies, thereby inhibiting photosynthesis and disturbing the oxygen balance of aquatic ecosystems [15–17]. Under anaerobic environmental conditions, azo dyes may undergo reductive cleavage of the azo bond, generating aromatic amines such as benzidine and aniline that are known for their carcinogenic and mutagenic properties [18–20]. These toxic intermediates can accumulate in aquatic organisms and may enter the food chain, posing long-term risks to both environmental and human health. Chronic exposure to such compounds has been associated with hepatotoxicity, reproductive toxicity, endocrine disruption, and an increased risk of cancer [21–25].

Several physicochemical treatment methods—including adsorption, advanced oxidation processes, membrane filtration, and coagulation–flocculation—have been widely employed for the removal of dyes from industrial wastewater. While these technologies can reduce dye concentration effectively, they often involve high operational costs, generate large quantities of secondary sludge, and frequently fail to achieve complete mineralization of the dye molecules [26–28]. These limitations have intensified the search for more sustainable, cost-effective, and environmentally compatible remediation technologies.

In this context, microbial bioremediation has emerged as a promising strategy for the detoxification and removal of azo dyes from contaminated environments. Microorganisms possess diverse enzymatic systems capable of catalyzing the reductive cleavage of azo bonds and the subsequent transformation of dye intermediates into less toxic products such as CO₂, H₂O, and NH₃ [29, 30]. Various microbial groups—including bacteria, fungi, yeasts, algae, and cyanobacteria—have demonstrated significant potential for azo dye degradation through the activity of key enzymes such as azoreductases, laccases, peroxidases, and other oxidoreductases [14, 31]. In addition, microbial consortia can further enhance dye degradation efficiency through synergistic metabolic interactions and cooperative enzymatic pathways under diverse environmental conditions [32, 33]. Recent advances in biotechnology—including genetic engineering, nanobiotechnology, immobilized biocatalysts, and advanced bioreactor configurations—have further improved the robustness, efficiency, and scalability of microbial dye remediation processes.

Despite the considerable progress achieved in microbial azo dye degradation, several critical challenges still hinder the practical implementation of these systems in industrial wastewater treatment. Many reported microbial processes achieve efficient decolorization but fail to ensure complete mineralization of aromatic amine intermediates, which may retain significant toxicity. Furthermore, the performance of dye-degrading microorganisms often declines under real textile effluent conditions characterized by high salinity, fluctuating pollutant loads, mixed dye compositions, and the presence of auxiliary chemicals used during textile processing. Another important limitation is the relatively limited integration of metabolic engineering and systems-level approaches to optimize microorganisms and microbial consortia for efficient dye degradation. Addressing these challenges requires a deeper understanding of microbial degradation pathways, enzyme regulation, and metabolic interactions among microbial communities. Therefore, this review critically examines recent progress in microbial azo dye biodegradation, with particular emphasis on enzymatic mechanisms, microbial consortia, and emerging biotechnological strategies that enhance the efficiency, stability, and sustainability of microbial systems for dye remediation.

## Toxicological concerns of azo dyes and their degradation products

### Impact of azo dyes on aquatic ecosystems

Azo dyes represent a significant class of environmental pollutants due to their extensive use in textile and related industries, combined with their high chemical stability and resistance to natural degradation processes [11]. The discharge of wastewater containing azo dyes—often untreated or insufficiently treated—can significantly disrupt the structure and functioning of aquatic ecosystems [26, 34]. One of the most evident environmental impacts of azo dyes is the reduction of light penetration in aquatic systems. Even at relatively low concentrations, their intense coloration limits sunlight transmission through the water column, thereby inhibiting photosynthesis in algae and submerged plants. This disruption ultimately reduces dissolved oxygen levels and interferes with primary productivity within aquatic ecosystems [15]. This phenomenon is often described as the “shade effect,” referring to the visual and ecological consequences of light attenuation caused by dye contamination.

Azo dyes can also increase both biological oxygen demand (BOD) and chemical oxygen demand (COD) in contaminated water bodies due to their complex organic structures and resistance to rapid degradation. During microbial attempts to degrade these complex molecules—often inefficiently—substantial amounts of dissolved oxygen are consumed. This process may lead to hypoxic or anoxic conditions, especially in stagnant or slow-moving water bodies, which in turn harm the survival of fish, amphibians, and other aquatic organisms that are aerobic [16, 17]. A study investigating the effects of azo dyes on zebrafish reported severe developmental toxicity, including hatching difficulties and genetic abnormalities such as cardiac swelling, reduced heart rate, placental edema, and spinal deformities at concentrations ranging from 5 to 50 mM. The dyes were also completely lethal to embryos at a concentration of 100 mM [35]. Moreover, many azo dyes undergo reductive cleavage in anaerobic sediments, which results in aromatic amines that are highly toxic and persistent. These compounds may accumulate in aquatic organisms, alter the activity of enzymes, impair endocrine functions, and cause cellular damage. Fish exposed to certain azo colours have shown reduced growth, reproductive failure, gill damage, and increased mortality. Moreover, these effects may not be limited to individual organisms but extend to impacts at the level of society, such as reduced biodiversity and altered dynamics of food webs [36, 37]. Therefore, the continuous release of azo dye–containing effluents can significantly deteriorate water quality and contribute to long-term ecological imbalances that threaten the stability and sustainability of freshwater and marine ecosystems.

### Impact of azo dyes on soil and microbial construction

Azo dyes and their degradation products can significantly alter soil physicochemical properties and disrupt the balance of microbial communities that are essential for nutrient cycling, organic matter decomposition, and plant productivity, particularly in areas irrigated with or exposed to industrial wastewater [34]. One of the primary consequences of azo dye contamination is the reduction in both the diversity and metabolic activity of soil microbial populations. Soil microorganisms, including bacteria and fungi, are particularly sensitive to synthetic organic pollutants. Exposure to azo dyes can suppress beneficial microbial populations, reduce enzymatic activity, and promote the emergence of resistant or opportunistic microbial species. For example, a study on Reactive black 5 contamination found that at low dye concentrations, microbial biomass and soil respiration were not affected, but litter degradation and nitrogen mineralisation were affected [38, 39].

The extent of these harmful effects is largely determined by the molecular structure, chemical reactivity, and nature of the substituent groups present in the dyes. Among the most problematic degradation products are aromatic amines, which are highly persistent and often resistant to conventional wastewater treatment processes. These compounds often remain in the environment longer than the parent dyes and, at elevated concentrations, can inhibit microbial activity essential for dye degradation due to their own toxicity [40, 41]. Soils contaminated with azo dyes often exhibit altered physicochemical properties. High pH and electrical conductivity are common in contaminated soils, which may harm soil fertility [34, 39]. In addition, a decrease in organic matter and water retention capacity was reported, further reducing the productivity of the soil [42]. Azo-based pollution has been shown to interfere with key soil biogeochemical processes, particularly those involved in the turnover of nitrogen. For example, Reactive Black 5, a widely used azo dye, has been shown to inhibit the oxidation of ammonium and thus to reduce the conversion of ammonium to nitrate. Such inhibition can have cascading effects on soil fertility because nitrates represent a major bioavailable nitrogen source for plants [43].

### Carcinogenic and mutagenic effects on human health

In humans, exposure to azo dyes has been associated with a wide range of adverse health effects, including neurotoxicity, cardiotoxicity, pulmonary dysfunction, reproductive disorders, allergic reactions, mutagenicity, and carcinogenicity [44]. Human exposure may occur through multiple routes, including dermal contact, inhalation of dye-containing dust, and ingestion of contaminated food or water, potentially resulting in acute or chronic toxic effects. Although azo dyes are relatively stable in their parent form, they can undergo reductive degradation under anaerobic or microbial conditions, particularly in the gastrointestinal tract following occupational exposure in textile, printing, leather, and dye industries. This reductive cleavage generates aromatic amines, a class of compounds widely recognized for their carcinogenic and mutagenic properties [21–23, 45].

#### Carcinogenic effects of azo dyes

Azo dyes have been linked to an increased risk of cancer due to their ability to form reactive intermediates that interact with DNA. These interactions can lead to mutations in critical genes involved in cell cycle regulation, ultimately resulting in tumor formation. For instance, benzidine-based azo dyes commonly used in textile applications have been identified as particularly hazardous due to their strong association with carcinogenic aromatic amine metabolites [46]. Epidemiological studies have associated long-term exposure to azo dyes with an increased incidence of various cancers, including liver, bladder, and colon cancers. Epidemiological studies have associated long-term exposure to certain azo dyes with an increased incidence of cancers, particularly those affecting the liver, bladder, and colon. Occupational exposure in dye manufacturing and textile industries has been strongly linked to elevated bladder cancer incidence among workers, highlighting the carcinogenic potential of specific azo dyes and their degradation products [24]. In experimental studies, oral administration of azo colouring agents such as carmoisine has been associated with hepatotoxicity and hepatic oncogenesis in mice [25]. These findings indicate that high-dose exposure to certain azo dyes may induce renal dysfunction and hepatic tumor formation, further highlighting their carcinogenic potential.

#### Mutagenic effects of azo dyes

Azo dyes can induce genetic mutations through DNA damage, leading to chromosomal aberrations and micronucleus formation [47]. For example, a study by Bienstock et al. [48] used molecular dynamics simulations to investigate how Sudan I and Sudan II, and their metabolites, interact with DNA. Their results indicated that Sudan I and Sudan II can form DNA adducts, causing structural alterations in the DNA helix that interfere with replication and repair processes and ultimately contribute to genotoxic effects. The mutagenicity of 397 aromatic amines potentially released from 470 known textile azo dyes has also been investigated. The study identified 40 mutagenic aromatic amines that may originate from the cleavage of approximately 180 different parent azo dyes, highlighting a greater concern regarding the mutagenic potential of azo dye degradation products than previously anticipated [47].

**Fig. 2:**
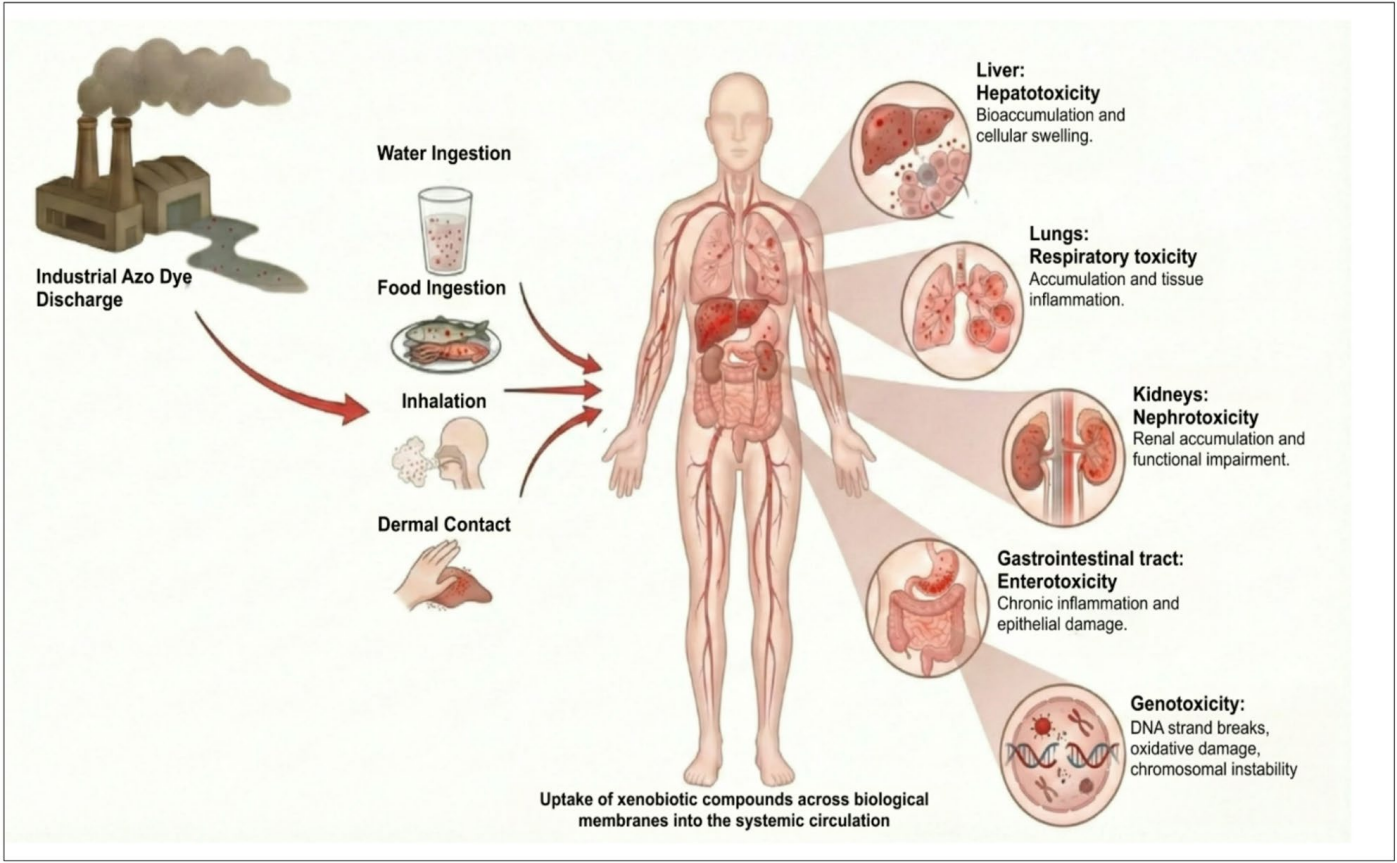
Environmental exposure pathways and potential toxicological effects of azo dyes in humans. Industrial discharge of azo dye–contaminated effluents into aquatic environments can lead to human exposure through water ingestion, contaminated food consumption, inhalation of dye particles, and dermal contact. After entering the body, these xenobiotic compounds may cross biological membranes and circulate systemically, potentially causing organ-specific toxicities including hepatotoxicity, respiratory toxicity, nephrotoxicity, gastrointestinal inflammation, and genotoxic effects

In addition to their mutagenic potential, aromatic amines are known to induce a variety of adverse health effects, including urinary tract disorders, hematopoietic abnormalities, porphyria, and hypersensitivity reactions. At elevated exposure levels, these compounds may trigger severe allergic reactions, including Stevens–Johnson syndrome and toxic epidermal necrolysis (Lyell’s syndrome), both of which are life-threatening dermatological conditions [21]. Recent toxicological investigations have further clarified the health risks associated with azo dyes and their degradation products. Studies using human cell lines and relevant animal models have evaluated the biological effects of azo dye degradation products. The major degradation products of various azo dyes and their associated toxicological effects on human health are summarized in Table 1. The primary exposure and accumulation pathways and their potential multi-organ impacts during prolonged exposure are illustrated in Fig. 2.

**Table 1:** Toxic effects of azo dyes and their degradation products on human health

Azo dye type	Degradation product(s)	Observed effects	References
Disperse Yellow 7	4-Aminobiphenyl, p-Phenylenediamine	DNA damage, liver carcinogenicity, and mutagenicity	Balakrishnan et al. [49]
Disperse Red 1	Nitrobenzene, 4-Nitrobenzamine, 2-(Ethylphenylamino)-ethanol	mutagenic in different cell systems	Chequer et al. [50]
Disperse Orange 1	2-Nitroaniline	DNA damage and apoptosis in human hepatoma (HepG2) cells	Ferraz et al. [51]
Disperse Blue 165, Disperse Red 73	2-Cyano-4-nitroaniline, 2,6-Dicyano-4-nitroaniline	Potent frameshift mutations in the Ames test, enhanced by nitroreductase and acetyltransferase	Josephy et al. [52]
Direct Blue 14	o-Tolidine (3,3’-dimethylbenzidine)	Metabolized by human skin bacteria to carcinogenic aromatic amines	Platzek et al. [53]
Direct Red 28	Benzidine, 4-Aminobiphenyl	DNA damage and apoptosis in HL-60 human leukemia cells during initial biodegradation stages	Bafana et al. [54]
Sudan I	Benzenediazonium ion	Liver and urinary bladder carcinogenicity; DNA adduct formation	Bienstock et al. [48]
Sudan II	Benzenediazonium ion	Bladder carcinomas; DNA damage	Bienstock et al. [48]
Amido Black 10B	p-Nitroaniline	Induced DNA damage in prokaryotic and eukaryotic cells	Cruz Brambilla et al. [55]
Acid Orange 7	1,2-Naphthoquinone, 1,4-Benzoquinone	Initial degradation leads to toxic aromatic compounds	Le et al. [56]
Acid Red G	Not available	Cytotoxicity in HeLa and human lymphocyte cells	Faldu et al. [57]
Tartrazine	Not available	Induced significant DNA damage in HaCaT, A549, and HepG2 cell lines	Zand et al. [58]
Tartrazine E102	Sodium 2-aminobenzenesulfonate	Elevated liver enzymes (AST, ALT, ALP) with hepatocyte apoptosis, mild hepatic and renal inflammation, and tubular necrosis	Alshehrei, [59]
Tartrazine (E102, FD&C Yellow 5)	Sulfanilic acid, 4-Amino-3-carboxy-5-hydroxy-1-(4-sulfophenyl)pyrazole, Purpurazoic acid	SCAP and PPA are moderately toxic to human cells. These compounds can affect human cell viability at relatively low concentrations	Pay et al. [60]
Reactive Black 5	3,4,6-triamino-5-hydroxynaphthalene-2,7-disulfonate, 3,6,8-triaminonaphthalen-1-ol	Cytotoxicity in human cell lines (MTT assay)	Bilal et al. [61]
Reactive Black 5	Naphthalene-1,2-diamine, 4-(methylsulfonyl) aniline	Ecotoxicity is observed in aquatic organisms	Lumbaque et al. [62]
Reactive Yellow 84	Not available	May cause oxidative stress and DNA damage	Zhang et al. [63]
Reactive Green 19	Not available	Induces cytotoxicity, oxidative stress, and inflammation	Leme et al. [64]
Brown HT (E155)	Not available	Hepatotoxicity, renal impairment, and histopathological changes in liver and kidneys	Islam et al. [65]

### Bioaccumulation and biomagnification

Although some azo dyes exhibit relatively low acute toxicity in their parent form, their persistence, lipophilicity, and slow degradation rates enable them to accumulate in biological tissues over time, resulting in chronic exposure and delayed toxic effects [34, 66]. In contaminated environments, azo dyes and their metabolites may progressively accumulate in the tissues of exposed organisms, particularly in lipid-rich tissues and organs such as the liver and kidneys. Numerous studies have demonstrated that fish, shellfish, and other aquatic organisms can accumulate azo dyes and their degradation products through direct exposure to contaminated water or through the ingestion of polluted food sources [45, 56].

**Fig. 3:**
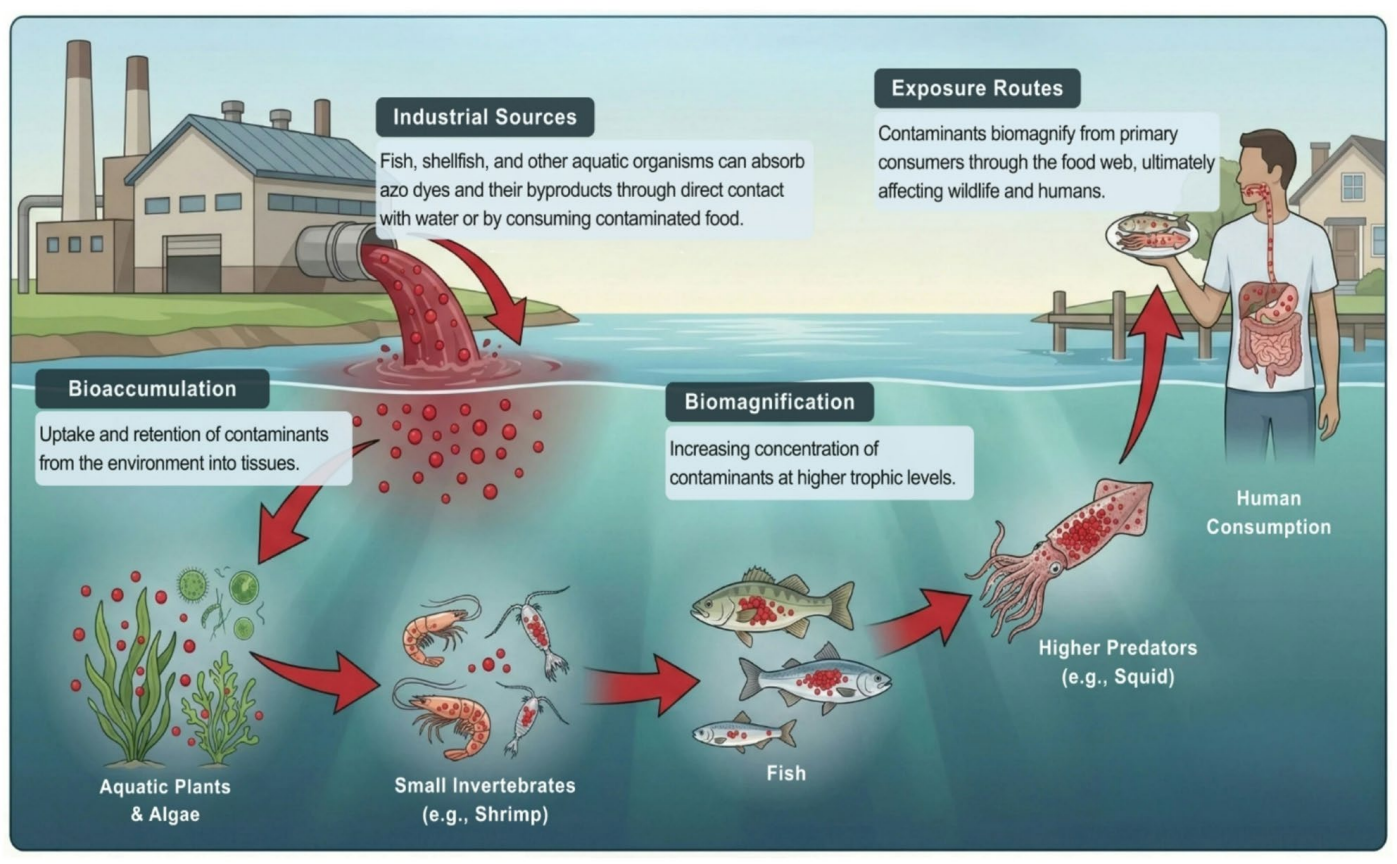
Bioaccumulation and biomagnification of azo dyes and their degradation products in aquatic ecosystems. Industrial discharge of dye-contaminated effluents introduces pollutants into water bodies, where they are absorbed by aquatic plants and algae and subsequently transferred through the food chain to invertebrates, fish, and higher predators. The progressive accumulation and trophic transfer of these contaminants ultimately lead to human exposure through the consumption of contaminated aquatic organisms

For example, aromatic amines produced from the reductive cleavage of azo bonds may accumulate in fish liver and muscle tissues, posing potential risks to human consumers through the food chain. In contrast, biomagnification refers to the progressive increase in the concentration of toxic substances as they move through successive trophic levels of the food chain. When primary consumers such as zooplankton or small fish ingest contaminated algae or sediment, these toxic compounds can accumulate within their tissues. As predators consume contaminated prey, the concentration of these dyes or their metabolites progressively increases, resulting in greater toxic loads in higher trophic organisms such as birds, mammals, and ultimately humans ([45]; [16]). The environmental entry routes of azo dyes and their major exposure pathways are illustrated in Fig. 3. Increasing evidence indicates that residues of azo dyes and their degradation products can accumulate in edible plants irrigated with contaminated water and in livestock exposed to polluted drinking sources. This raises significant concerns regarding indirect human exposure through contaminated food products, potentially leading to chronic toxicity, genotoxic effects, and increased carcinogenic risk [66, 67].

## Conventional physiochemical processes for treating azo dyes

Water containing azo dyes is a problematic issue that must be treated. However, unsuitable treatment can damage the environment and human health [28]. Several methods have been developed to remediate dye effluent in recent years, including chemical, physical and biological routes. Physiochemical options, such as adsorption, membrane filtration, ion exchange, advanced oxidative process and coagulation-flocculation method [68].

### Adsorption

Adsorption is surface based process in which attraction occurs between adsorbates (liquid molecules), and adsorbents (solid surface) to remove dyes. This process depends on adsorbents that contain pores in their structure to increase surface area, leading to an increase in the separation efficiency [26, 32]. On the basis of how adsorption occurs between adsorbates and adsorbents, the adsorption process is divided into physical adsorption and chemical adsorption. At physical adsorption, the adsorbate and adsorbent are connected together by weak bonds like vander waals forces, hydrogen bonding, and electrostatic interactions. This process is considered a reversible adsorption. In chemical adsorption, the adsorbate and adsorbent are connected together by strong bonds like covalent and ionic bonds. This process is considered an irreversible adsorption [69]. Activated carbon is the most common material used as the adsorbent, but it is limited in application due to its high cost, and the reuse of activated carbon is not a simple process. Some alternatives for activated carbon are used, such as minerals, silica gel and agricultural wastes (peat, wood chips, wheat straw, and corn cob). They are low-cost but have poor separation efficiency. Chitosan is also used as an adsorbent, which is derived from chitin as a modified natural biopolymer [70]. Numerous recent studies aim to use hydrogel, metal–organic frameworks and polypyrrole for separation [32]. The adsorption process makes removal in a short time with high efficiency, but it is an expensive process.

### Membrane filtration and ion exchange

Membrane filtration technique is used for the removal of chemical pollutants and dyes, but not for their degradation, as it reduces the dye concentration in industrial wastewater. By using a small-pore membrane, we can remove suspended solids and unwanted materials, which have a size larger than the membrane pores, to produce dye free solution. Membrane filtration is anaerobic pre-treatment followed by aerobic and membrane post- treatment [27, 35, 71]. Microfiltration (MF), Nanofiltration (NF), Ultrafiltration (UF), and reverse osmosis (RO) are used for the removal of dyes. MF is a process that uses a membrane with pores size range from 0.1:10 micrometer to remove dye and suspended solids. MF is used as pre-treatment for reverse osmosis and nanofiltration. NF is an advanced technique that has been used to remove dyes and low molecular weight compounds from textile wastewater, and membrane pores size range from 0.2 to 0.5 nm, so by size and electrostatic repulsion mechanism, dyes can be removed from wastewater [72]. Adsorption is used with filtration to reduce dye concentration and increase efficiency. NF is rarely used due to salt accumulation. UF is used for organic dye removal, but dye removal is in the range from 31% to 76% only. Membrane diameter ranges from 0.1 to 0.001 microns. It is low cost method, but the separation time is long due to the large pore size. RO is a method used to remove all mineral salts, ions, chemical pollutants and reactive dyes to produce high water quality. RO include degradation and elimination of chemical pollutants in one step. By increasing salt concentration in water, the size required for separation increases [26, 69]. Membrane filtration methods have some advantages, including simplicity, smaller space, and a short time for separation. However, it is limited because of membrane fouling, great energy costs, scaling issues, producing secondary toxic sludge and the final product quality must be checked [71].

The ion exchange is an effective technique for the treatment of all categories of dyes and for wastewater containing high concentrations of metal ions due to its benefits, such as low cost, simplicity, flexibility, and regeneration. Although it has wide applications, there are some limitations associated with the ion exchange technique, as it produces a highly concentrated waste that needs careful disposal, and it can only handle a narrow spectrum of dyes. The mechanism of ion exchange depends on the strong interactions between functional groups of a solid matrix and charged dyes [73].

### Advanced oxidative process

Advanced oxidation processes (AOPs) rely on the generation of highly reactive species, particularly hydroxyl radicals (•OH), which are capable of rapidly oxidizing complex organic pollutants. Hydroxyl radicals act as powerful oxidizing agents capable of degrading azo dyes through oxidative cleavage of aromatic structures and chromophoric groups [74]. Several AOP-based technologies have been developed for dye remediation, including photocatalysis, Fenton and photo-Fenton reactions, electrochemical oxidation, and ozonation. AOPs are widely regarded as efficient treatment strategies because they can rapidly oxidize dye molecules and convert them into smaller, less complex compounds such as CO₂ and H₂O. Despite these advantages, AOPs present several limitations, including high operational costs, significant energy requirements, pH sensitivity, and the potential formation of secondary sludge or toxic intermediates [75, 76]. Among AOP technologies, photocatalysis has been extensively investigated for the treatment of textile wastewater due to its ability to generate reactive radicals under light irradiation [77]. Semiconductor photocatalysts such as ZnO and TiO₂ generate electron–hole pairs under light exposure, producing reactive species including hydroxyl and superoxide radicals that promote dye degradation. These systems can operate under solar irradiation, making photocatalysis an attractive and potentially energy-efficient treatment strategy [78, 79]. The Fenton reaction is another widely applied AOP for degrading organic pollutants, including azo dyes and other recalcitrant contaminants. This process generates hydroxyl radicals through the reaction between hydrogen peroxide and ferrous ions (Fe²⁺), commonly referred to as Fenton’s reagent. However, the Fenton process operates effectively only under acidic conditions and may generate considerable amounts of iron-containing sludge as secondary waste [27, 80]. Ozonation employs ozone (O₃) as a strong oxidizing agent capable of attacking dye chromophores and breaking down complex molecular structures. While in the presence of non-soluble dyes, O_3_ undergoes slow decolourization. Under alkaline conditions, ozone decomposition generates hydroxyl radicals that enhance oxidation efficiency and promote the formation of smaller biodegradable compounds. This method has some limitations, such as high cost of O_3_ production, short half-life of O_3_, and its unstable nature makes it undesirable for wastewater treatment [81]. Electrochemical oxidation has also emerged as a promising AOP-based technique for the treatment of dye-contaminated wastewater [82]. Common electrochemical approaches include anodic oxidation, electrocoagulation, and electro-Fenton systems. These systems can operate without the addition of external chemical reagents and may generate minimal sludge. Nevertheless, their large-scale application may be constrained by high electricity consumption and operational costs [83, 84].

### Coagulation–flocculation

Coagulation–flocculation followed by sedimentation is widely used to remove sulfur dyes, disperse dyes, reactive dyes, and other organic pollutants from wastewater. In this technique, coagulants such as metal salts neutralize the electrical charge of suspended particles, whereas flocculants—typically polymeric compounds—promote the aggregation of destabilized particles into larger flocs that can be easily separated. Coagulants are added during the vigorous mixing stage. After neutralization, the suspended solids can collide to form microflocs and the flocculants are mixed with these microflocs to form large particles called macroflocs that can be easily separated by sedimentation [35, 85]. Common chemical coagulants such as ferric chloride, ferrous sulfate, and ferric sulfate are widely used to enhance pollutant removal efficiency. Other coagulants, including magnesium carbonate and hydrated lime, can facilitate the adsorption and precipitation of azo dyes and their degradation products. Recent studies use natural and synthetic polymers as modified chitosan-based flocculants and dextran-based flocculants for highly efficient removal [86, 87]. Although this method is economical and operationally simple, its application may be limited by pH sensitivity and the generation of chemical sludge that requires further treatment or disposal [88].

## Bioremediation of textile azo dyes

The treatment of dye-contaminated effluents has become a major environmental challenge. Although various conventional physicochemical methods have been applied to remove dyes from textile effluents, these approaches present several limitations, including the formation of toxic byproducts, high energy or chemical requirements, elevated operational costs, and incomplete mineralization of azo dyes [7, 89, 90]. In contrast, bioremediation has emerged as a promising alternative strategy for the detoxification and removal of hazardous pollutants such as azo dyes from contaminated environments. Unlike conventional methods, it is cost-effective, minimizes harmful byproducts, and often achieves complete mineralization of dyes into CO₂ and H₂O. In addition, it is more economical and more sustainable, making it highly suitable for wastewater treatment [29, 30]. Consequently, bioremediation represents a promising and environmentally responsible strategy for the long-term management of dye-contaminated wastewater, offering advantages that align with both regulatory requirements and sustainable development goals. Numerous microorganisms, including bacteria, fungi, yeasts, algae, and cyanobacteria, have demonstrated the ability to degrade a wide range of azo dyes [91]. Bioremediation can be achieved via several mechanisms, including enzymatic degradation, biosorption, bioaccumulation, co-metabolism, and the simultaneous operation of these processes [16, 31].

### Mechanisms of azo dye bioremediation

#### Enzyme–mediated bioremediation

**Fig. 4:**
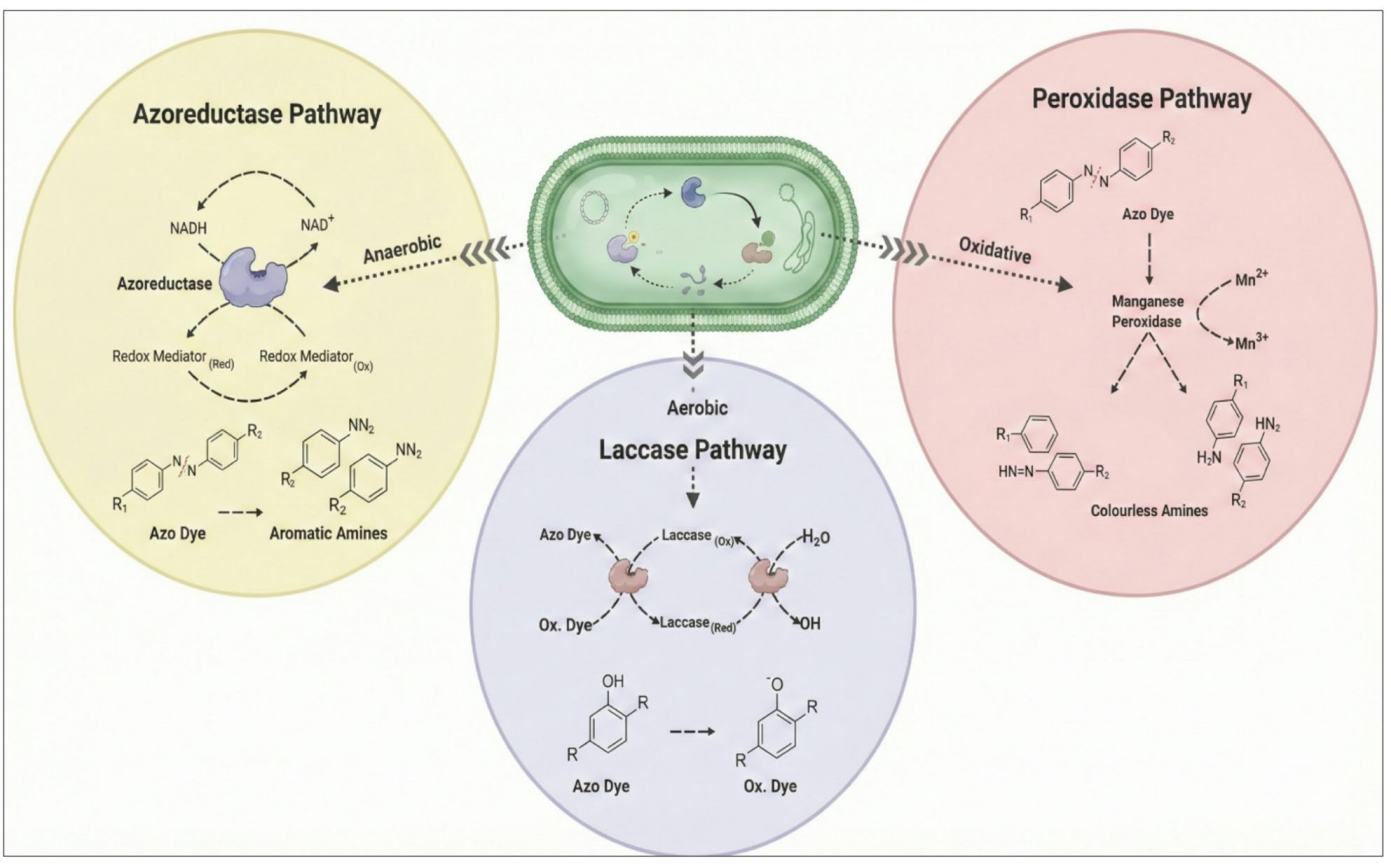
Schematic representation of major microbial enzymatic pathways involved in azo dye biodegradation. Under anaerobic conditions, azoreductase catalyzes the reductive cleavage of azo bonds (–N = N–) using NADH-dependent electron transfer, producing aromatic amines. Under aerobic conditions, oxidative enzymes such as laccases and peroxidases further transform dye molecules and their intermediates through radical-mediated oxidation reactions, ultimately leading to dye decolorization and degradation

Enzymatic bioremediation involves the use of microbial enzymes to catalyze the breakdown of complex and recalcitrant pollutants into simpler, less toxic, and more biodegradable compounds [92]. The resulting aromatic amine intermediates are subsequently transformed and mineralized into simpler compounds such as carbon dioxide, water, and ammonia, which can then be assimilated into microbial metabolic pathways to support cellular respiration and biomass synthesis [14]. The most common oxidoreductive enzymes involved in dye degradation include reductases such as azoreductase and NADH–DCIP reductase, as well as oxidases and peroxidases including laccase, tyrosinase, lignin peroxidase, and manganese peroxidase [93, 94]. Azoreductase and nicotinamide adenine dinucleotide–dichlorophenol indophenol (NADH–DCIP) reductase are among the most extensively studied enzymes involved in dye degradation, mediating azo dye reduction via electron transfer from reducing cofactors such as NADH and NADPH to the dye substrates (Fig. 4) [95].

Several mechanisms have been proposed to explain the exact enzymatic mechanism of azo dye reduction. For instance, in flavin-dependent azoreductase, reduced flavins (FMN or FAD) act as intermediates in electron transfer from cofactors such as NADH or NADPH to the azo bonds. The process begins with NADH or NADPH donating electrons to the flavin prosthetic group, reducing it to FMNH₂ or FADH₂. These reduced flavins then transfer electrons directly to the azo compound, breaking the –N = N– bond and ultimately converting the dye into amine products [96]. This process occurs primarily under oxygen-limited or anaerobic conditions because oxygen competes as a stronger electron acceptor than azo dye molecules, thereby inhibiting efficient reductive cleavage and dye decolorization [30].

Similarly, NADH–DCIP reductase activity has been reported to increase in activity during the biodegradation of various azo dyes by strains of *Pichia occidentalis*, *Bacillus* spp., and *Cyberlindnera samutprakarnensis* (Ashar et al. 2022); [97, 98]). Interestingly, although azoreductase and NADH–DCIP reductase can jointly contribute to azo dye degradation in many cases, NADH–DCIP reductase may exhibit salt-tolerant behavior and preferential activation in halotolerant or halophilic microflora, whereas azoreductase tends to function preferentially under low-salinity conditions within the same microorganism [99].

While azoreductase and NADH–DCIP reductase mediate the initial step of azo bond reduction, other enzymes, including laccase, peroxidases, and tyrosinase, contribute to further degradation, ultimately leading to the complete mineralization of the dye [100]. Nonetheless, none of these enzymes participate in the initial cleavage of the azo bond except azoreductase, highlighting its crucial role despite its low secretion levels [101]. In addition, both oxidases and peroxidases require oxygen and hydrogen peroxide, respectively [102, 103]. Therefore, the incorporation of both aerobic and anaerobic conditions, either in sequential steps or as separate conditions, provides an effective strategy for enhancing azo dye biodegradation [104–106], consequently achieving more complete mineralization of dyes into CO₂ and H₂O [29, 30].

#### Biosorption and bioaccumulation–mediated bioremediation

In contrast to enzymatic degradation, biosorption and bioaccumulation involve the removal of dye molecules through their direct interaction with microbial cells [107]. In biosorption, dyes are adsorbed onto the surface of microbial biomass—whether living or dead—through various physicochemical mechanisms, including electrostatic attraction, hydrogen bonding, ion exchange, and van der Waals forces [108, 109]. The microbial cell wall provides multiple functional groups, such as hydroxyl and carboxyl moieties, that serve as active binding sites for dye adherence [110]. In contrast, bioaccumulation is an active, metabolism-dependent process in which living cells internalize dye molecules through membrane transport systems, leading to their sequestration within intracellular compartments [111, 112]. Both processes play a vital role in dye removal and can operate synergistically with enzymatic degradation to enhance the overall efficiency of bioremediation [107, 113].

#### Bioflocculant–mediated bioremediation

Bioflocculants are extracellular polymeric substances (EPS) produced by microorganisms—including bacteria, algae, fungi, and actinomycetes—through secretion or cell lysis, and they can also be obtained from sources such as activated sludge and palm oil mill effluents [114, 115]. These bioflocculants, primarily composed of polysaccharides, proteins, or glycoproteins, act as natural binding agents that promote the aggregation of suspended particles or dye molecules into larger flocs, thereby facilitating their efficient removal from aqueous solutions in an eco-friendly and sustainable manner [116]. Unlike chemical flocculants, bioflocculants are biodegradable, non-toxic, and environmentally friendly, making them a sustainable option for wastewater treatment and dye decolorization [117, 118].

Bioflocculation occurs through several complementary mechanisms. First, adsorption bridging involves microbial flocculants linking colloidal particles via active sites, forming three-dimensional flocs stabilized by hydrogen bonds and van der Waals forces. Second, charge neutralization reduces the Zeta potential of negatively charged colloids, promoting aggregation. Third, chemical interactions between flocculant functional groups and particle surfaces further enhance floc formation. Fourth, volume sweeping allows growing flocs to capture additional dispersed particles during sedimentation. Together, these processes enable microbial flocculants to efficiently aggregate and remove suspended particles, including dyes, from aqueous solutions [119].

### Microorganism-mediated bioremediation of azo dye

#### Bacterial–mediated bioremediation

Bacterial remediation has been one of the most important biological approaches to wastewater treatment, since bacteria are highly diverse, adaptable, and capable of degrading a wide range of organic and inorganic contaminants. Bacteria have been shown to degrade azo dyes under aerobic, microaerophilic, and anaerobic conditions, either as isolated strains or within microbial consortia, and can do so in the presence of diverse carbon and nitrogen sources across broad ranges of pH, temperature, salinity, and other physicochemical parameters [14]. Bacterial mechanisms for the remediation of azo dyes include biosorption and enzymatic degradation. Biosorption encompasses both adsorption and absorption processes and is closely linked to the composition of lipids and heteropolysaccharides in the microbial cell wall. The presence of various charged functional groups in these components facilitates electrostatic interactions with azo dyes [120, 121]. However, bacterial enzymatic degradation relies on intra- and extracellular enzymes produced by bacterial cells to biodegrade azo dyes. The initial step in decolorization typically consists of the reductive cleavage of the azo bonds in the chromophoric groups, a reaction primarily catalyzed by azoreductases under anaerobic or low-oxygen conditions. The resulting aromatic amines, which are often toxic, are then further transformed predominantly into less toxic compounds through oxidative processes. Enzymes such as laccases and various peroxidases play a central role in these subsequent steps, facilitating the oxidation and breakdown of aromatic amines and other intermediates, ultimately enhancing bacterial detoxification and mineralization of the dye compounds [14, 122].

Numerous studies have documented the successful use of bacteria in azo dye bioremediation, as shown in Table 2. For instance, *Aliiglaciecola lipolytica* achieved approximately 90% decolorization of Congo Red through a combination of biosorption and enzymatic degradation [123]. Similarly, different bacterial species were able to decolorize eight different azo dyes via enzymatic pathways involving azoreductases and phenol oxidases, reaching decolorization efficiencies of up to 96.5% in some cases [124]. Interestingly, marine exoelectrogenic bacteria—microorganisms capable of extracellular electron transfer and thriving in saline environments—represent an emerging class of halotolerant/halophilic exoelectrogenic decolorizers (HEDs) for textile wastewater treatment [125]. In a recent study, *Shewanella marisflavi* EP1 demonstrated the ability to decolorize the azo dye Xylidine Ponceau 2R under extreme conditions, tolerating NaCl concentrations up to 20%. At lower salinities, intracellular reductive degradation predominates, whereas at higher salinities a bioflocculation mechanism takes over, in which extracellular polymers bind and aggregate dye molecules, facilitating their removal [126]. These findings underscore that different bacterial strains may utilize distinct biotransformation pathways, and the dye degradation depends not only on the microbial species but also on the chemical structure and position of functional groups within the dye molecule itself.

**Table 2:** Examples of bacterial bioremediation of azo dyes

Bacterial culture	Species/strain	Type of azo dye	Decolorization efficiency	Experimental conditions	Incubation time	References
Individual culture	*Providencia rettgeri*	Brilliant Crocein	≈ 94%	35 °C, pH 8, 200 mg/L Ethanol (Co-substrate)	192 h	Shi et al. [32]
	*Alcaligenes aquatilis*	Synazol Red 6HBN	82%	37 °C, pH 7	96 h	Ajaz et al. [127]
	*Bacillus* sp. VITAKB20	Reactive Orange 16	97.5%	37 °C, Shaking, Calcium Alginate Immobilization	48 h	Pandey et al. [128]
	*Lysinibacillus* sp. KPB6	Reactive Blue 250	98.2%	37 °C, Shaking, Calcium Alginate Immobilization	48 h	Pandey et al. [128]
	*Shewanella marisflavi* EP1	Xylidine Ponceau 2R	≈ 100%	35 °C, pH 7, NaCl (0–20%)	22 h	Xu et al. [126]
	*Bacillus* sp.	Ponceau 4R	95%	35 °C, pH 7, Salinity 5 g/L	24 h	Masarbo and Karegoudar [129]
	*Anoxybacillus* sp.	Direct Black G	≈ 92%	55 °C, pH 7.2	48 h	Chen et al. [130]
	*Streptococcus* sp.	Red/ Blue Azo Dye	> 90%	37 °C, pH 7	72 h	Saxena et al. [131]
	*Pseudomonas stutzeri*	Acid Blue 113	86.2%	37 °C	96 h	Joshi et al. [132]
Consortia	Mixed bacterial consortium from lake sediments: *Ruficoccus amylovorans*,* Azotobacter beijerinckii*,* Hydrogenophaga luteola*,* Pelagibacter ubique*,* Porticoccus hydrocarbonoclasticus*	Methyl Orange, Congo-Red	> 90%	30 °C, pH 7.1	50 h	Dissanayake et al. [133]
	Mixed bacterial community derived from textile effluent sludge: *Enterococcus faecalis* and *Klebsiella variicola*	Reactive Red 198	≈ 99%	37 °C, pH 8	72 h	Eslami et al. [134]
	Mesophilic bacteria consortium isolated from textile wastewater: *Pseudoarthrobacter* sp. and *Gordonia* sp.	Reactive Black 5	≈ 100%	37 °C, pH 11, Lactose and NH₄H₂PO₄ (Carbon and Nitrogen Sources)	168 h	Eskandari et al. [33]
	Cold-adapted bacteria consortium from textile wastewater: *Stenotrophomonas* sp. and *Sphingomonas* sp.	Reactive Black 5	≈ 100%	25–30 °C, pH 9, glucose and NH₄H₂PO₄ (Carbon and Nitrogen Sources)	168 h	Eskandari et al. [33]

A major concern when employing bacterial processes for bioremediation—particularly those involving potentially pathogenic genera and species—concerns may arise regarding their potential biological impact upon introduction into the environment. To mitigate such risks, several strategies can be adopted. These include: (i) utilizing isolated and purified enzymes or other bacterial products capable of decolorizing contaminants without the presence of viable bacterial cells [135]; (ii) employing bacterial strains or consortia isolated from the contaminated site or ecologically similar environments to enhance their compatibility with the existing microbiota [136]; and (iii) applying genetic engineering techniques to develop bacterial strains with programmed cell death mechanisms that terminate metabolic activity once the target contaminant is no longer present [137–140].

#### Fungal–mediated bioremediation

Fungal treatment has recently gained prominence as one of the most viable alternatives to conventional approaches for wastewater remediation. This capability is primarily attributed to the ability of fungi to secrete extracellular enzymes that degrade a wide spectrum of complex compounds, including dyes, aromatic hydrocarbons, polychlorinated biphenyls, and pesticides. Additionally, their extensive hyphal surface area enhances biosorption, further contributing to their effectiveness [27, 141]. The potential of fungal species in azo dye degradation has been widely demonstrated (Table 3). For instance, *Aspergillus* sp. has shown remarkable effectiveness in degrading a wide range of azo dyes, including Congo Red, Reactive Red M8B, Reactive Green HE4B, Direct Black BT, Direct Orange RS, Direct Sky Blue FF, and Direct Blue GLL [142–145].

**Table 3:** Examples of fungal and yeast bioremediation of azo dyes

Fungal/Yeast culture	Species/Strain	Type of azo dye	Decolorization efficiency	Experimental conditions	Incubation time	References
Individual culture	*Candida tropicalis* SYF-1	Acid Red B	≈ 94%	30 °C, pH 7, 95.0 mT static magnetic field	12 h	Tan et al. [146]
	*Pseudocochliobolus verruculosus* NFCCI 3818	Solvent Yellow 2	98%	28 °C, 10% (v/v) ethylene glycol	192 h	Nikam et al. [147]
	*Sterigmatomyces halophilus* SSA-1575	Reactive Black 5	100%	30 °C, pH 5, static culture	24 h	Al-Tohamy et al. [148]
	*Oudemansiella canarii* Laccase	Congo Red	80%	30 °C, pH 5.5	24 h	Iark et al. [149]
	*Pichia kudriavzevii* SDG12	Reactive Black 5	≈ 100%	32 °C, pH 7.5, Glucose (5 g/L)	18 h	Gholizadeh-Balderlou et al. [150]
	*Bjerkandera adusta*	Direct Black 80	95.5%	24 °C, pH 5.5	48 h	Gugel et al. [151]
	*Penicillium chrysogenum* Lacccase	Acid Blue 113	98%	32 °C, pH 5.5, HOBT (1 mM)	24 h	Senthilvelan et al. [152]
	*Trametes pubescens* MB 89	Malachite Green	≈ 100%	30 °C, 60 ppm initial concentration	59.5 h	Ejaz et al. [153]
	*Aspergillus flavus*	Congo Red	> 90%	30 °C, pH 6, Sucrose & NaNO_3_ (C/N Sources), Ca^+ 2^, static culture	192 h	Ghanaim et al. [143]
Consortia	A plant and yeast consortium: *Fimbristylis dichotoma* and *Saccharomyces cerevisiae*	Rubine GFL	92%	30 °C, pH 7	96 h	Jadhav et al. [154]
	Mixed fungal species: *Aspergillus aculeatus (*KCHW-1*)* and *Cladosporium tenuissimum (*MNDS-3*)*	Reactive Orange 16/ R. Green 19/ Remazol Brilliant Blue R	≈ 95–98%	25 °C, pH 5, 140 rpm agitation, Sucrose ammonium nitrate (C/N Sources)	12–24 h	Modi et al. [155]
	Mixed fungal species: *Aspergillus niger*,* Aspergillus terrus*,* Aspergillus oryzae*,* Aspergillus fumigatus*	Direct Violet /Methyl Red	≈ 100%	28 °C, pH 5, 150 rpm agitation	72 h	El-Rahim et al. [156]
	A yeast consortium: *Sterigmatomyces halophilus* SSA-1575 and *Meyerozyma guilliermondii* SSA-1547	Reactive Black 5	96.1%	35 °C, pH 7, static conditions	120 h	Al-Tohamy et al. [157]

The bioremediation of azo dyes by fungi occurs through two primary mechanisms: biosorption and biodegradation/biotransformation [158–160]. In biosorption, dye molecules adhere to the surface of fungal biomass, with efficiency depending on the cell surface characteristics and functional groups [161, 162]. For instance, *Aspergillus niger* has demonstrated significant potential for the decolorization of the azo dye Congo red, with biosorption being among the primary mechanisms involved in the decolorization process [163]. In contrast, biodegradation/biotransformation of azo dyes depends mainly on enzymes responsible for breaking chemical bonds in chromophores and auxochromes, which vary with dye structure [141]. When biodegradation is complete, the process is termed mineralization, producing simpler and less harmful products such as H₂O, CO₂, NH₃, CH₄, H₂S, and PO₃. However, if organic compounds are not fully mineralized, the process is instead referred to as biotransformation [164]. For instance, *Pleurotus eryngii* can degrade azo dyes, including Reactive Black 5 and malachite green, by producing ligninolytic enzymes such as laccase, manganese peroxidase (MnP), and lignin peroxidase (LiP), which transform the dye’s toxic complex structure into a less toxic compound [165–167].

Importantly, the main lignin-modifying enzymes involved in azo dye biodegradation by fungi are lignin peroxidase (LiP), manganese peroxidase (MnP), and laccases. Unlike bacteria, fungi typically employ these enzymes to degrade azo dyes under aerobic conditions [68, 168, 169]. Laccases (multi-copper oxidases) can directly oxidize phenolic or aromatic amine groups, sometimes cleaving azo bonds in the process [170], whereas LiP and MnP generate reactive radicals via H₂O₂ that non-selectively break down complex structures [7]. In summary, fungi offer a highly effective alternative to conventional methods for treating azo dye–contaminated wastewater. By combining biosorption with enzymatic biodegradation, they remove or transform complex dye molecules, leading to decolorization, detoxification, and, in some cases, complete mineralization of azo dyes.

#### Yeast–mediated bioremediation

Any microorganism suitable for dye bioremediation should possess two essential characteristics: the ability to tolerate high concentrations of dyes in effluents and the capacity to transform these dyes into stable, non-toxic compounds. Yeasts demonstrate considerable potential in this regard [8]. However, the role of yeasts in the bioremediation of dye–contaminated wastewater remains far less explored than that of bacteria and filamentous fungi. Nevertheless, numerous studies underscore the significant potential of yeasts, demonstrating that various species can be effectively harnessed for the bioremediation of wastewater contaminated with synthetic dyes [171, 172]. Various ascomycetous yeast species have been reported to decolorize synthetic azo dyes (Table 3) through mechanisms such as biosorption, bioaccumulation, and enzymatic biodegradation or biotransformation. Some of these promising yeasts include *Saccharomyces cerevisiae*,* Candida tropicalis*,* Pichia kudriavzevii*, and *Issatchenkia orientalis* [150, 154]; Tan et al. [146, 173].

The enzymatic biodegradation of azo dyes in yeasts is primarily attributed to azoreductase enzymes. Because these enzymes require anaerobic or low-oxygen conditions to function effectively, ascomycetous yeasts have been extensively investigated for their presence and activity. Their strong fermentative capacity and facultative anaerobic metabolism make them particularly well-suited for sustaining the reductive environments necessary for azoreductase activity [174]. Moreover, ligninolytic enzymes such as laccase, tyrosinase, lignin peroxidase, and manganese peroxidase (MnP) have also been identified and studied in ascomycetous yeasts, further underscoring their capacity to biodegrade complex azo dyes [175, 176].

Basidiomycetous yeasts, despite belonging to a taxonomic group renowned for ligninolytic activity, have long been underestimated for their dye-removal potential. This is particularly surprising given the widespread occurrence of potent oxidative enzymes—such as laccase, manganese peroxidase (MnP), lignin peroxidase (LiP), and tyrosinase—in filamentous basidiomycetes [177]. Recent studies reveal that these yeasts utilize multiple mechanisms for azo dye removal, including biosorption (*Saccharomyces cerevisiae*) [178], bioaccumulation (*Leucosporidium muscorum*) [179], and enzymatic degradation (*Trametes trogii)* [180]. Collectively, these findings underscore the broader and more versatile decolorization capacity of basidiomycetous yeasts, which combine passive and active uptake with ligninolytic enzyme–driven breakdown of complex dye molecules.

#### Algal and cyanobacteria–mediated bioremediation of azo dye

Phycoremediation refers to the application of macroalgae, microalgae, and cyanobacteria for the removal and/ or biotransformation of environmental contaminants [181, 182]. Algae and cyanobacteria play a crucial role in the bioremediation of azo dye–contaminated wastewater, utilizing their photosynthetic and metabolic capacities to remove, transform, or immobilize pollutants [183, 184]. Numerous species have been identified for their potential in the removal of a wide variety of synthetic azo dyes (Table 4). The removal of azo dyes is achieved through a synergistic interplay of biosorption, bioaccumulation, and enzymatic degradation. The algal cell walls, enriched with polar functional groups (–OH, –COOH, –NH₂, –PO₄), provide multiple binding sites that capture dye molecules via electrostatic and hydrogen-bonding interactions [185]. Once bound, these dyes undergo enzymatic cleavage by key enzymes such as azoreductases and laccases, which break the characteristic azo bonds, ultimately transforming the dyes into less toxic or harmless end-products, including NH₃ and CO₂ [186].

**Table 4:** Examples of algal and cyanobacterial bioremediation of azo dyes

Algal/Cyanobacterial culture	Species/Strain	Type of azo dye	Decolorization efficiency	Experimental conditions	Incubation time	References
Individual culture	*Chlorella vulgaris*	Disperse Orange 2RL / Reactive Yellow 3RN	≈ 30–55%	25 °C, pH 6.8, Continuous illumination (80 µE/m^2^s)	168 h	El-Sheekh et al. [187]
	*Aphanocapsa elachista*	Disperse Orange 2RL / Reactive Yellow 3RN	≈ 26–50%	25 °C, pH 6.8, Continuous illumination (60 µE/m^2^s)	168 h	El-Sheekh et al. [187]
	*Spirulina platensis*	Acid Black 7 / Acid Black 210	≈ 97–99%	60 °C, pH 2, 125 ppm initial conc., biosorbent conc. 0.5 g/L	≈ 1 h	Al Hamadi et al. [188]
	*Oscillatoria* sp.	Malachite green / Methylene Blue / Safranin	93%	25 °C, Sunlight	120 h	Gelebo et al. [189]
	*Tetradesmus obliquus*	Acid Blue 92	≈ 95%	25 °C, pH 6.5	96 h	Ghadaki et al. [190]
	*Lychaete pellucida*	Reactive Blue 4 / R. Red 120 / R. Brilliant Yellow 3G / R. Green 12	95%	25 °C, pH 8, 5 ppm initial conc., biosorbent conc. 2 g/L	2 h	Khalaf et al. [191]
Consortia	Algal and cyanobacterial consortium: *Scenedesmus obliquus* and *Oscillatoria* sp.	Reactive Orange 122/ Reactive Red 194	> 97%	25 °C, pH 9–11	168 h	El-Sheekh et al. [183]
	Bacterial and algal consortium: *Pseudomonas putida*,* Chlorella* sp., *Lactobacillus plantarum*	Reactive Blue 40	99%	35 °C, pH 11, 150 rpm, agitation	144 h	Ayed et al. [192]
	Bacterial and algal consortium: *Enterobacter* sp. MN17 and *Chlorella vulgaris*	Azo dye-containing textile wastewater	70%	25 °C, pH 7, 150 rpm, agitation	48 h	Mubashar et al. [193]
	Mixed algal consortium: *Chlorococcum* sp. and *Scenedesmus obliquus*	Reactive Orange 122/ Reactive Red 194	89–91%	25 °C, pH 11, aeration	168 h	El-Sheekh et al. [194]

A study investigating the efficiency of algal species in azo dye bioremediation reported that *Shewanella* algae achieved a 95% removal of Acid Red 27 by cleaving the azo bond and reducing it to aromatic amines [195]. Likewise, the cyanobacterium *Aphanocapsa elachista* and the algal strain *Chlorella vulgaris* exhibited moderate azo dye removal, primarily attributed to the activity of the enzyme azoreductase, which plays a key role in catalyzing azo bond reduction [187]. Another notable example is the cyanobacterium *Arthrospira (Spirulina) platensis*, which has demonstrated exceptional dye adsorption capacity. Under optimal conditions, it removed more than 95% of Acid Black 210 and over 97% of Acid Blue 7, highlighting its strong potential for large-scale bioremediation of azo dyes [188].

Understanding and optimizing phycoremediation conditions is critical for achieving maximum dye removal efficiency. For example, *Chlorella vulgaris* removed 73–86% of the azo dye Maxilon Red in a batch photobioreactor, where dye-laden water is treated in a single load without inflow or outflow, resulting in gradually changing conditions such as pH, nutrient availability, and dissolved oxygen. By contrast, a continuous photobioreactor—maintaining a steady supply of dye-containing wastewater and nutrients while simultaneously removing treated effluent—achieved ~ 98% removal of the dye. This steady-state operation supports higher algal densities, more stable environmental parameters, and enhanced long-term decolorization performance compared to batch systems [185]. In summary, algal cells exhibit remarkable potential for the bioremediation of azo dye–contaminated wastewater. Their versatility under different environmental conditions, coupled with their ability to utilize sunlight and assimilate nutrients from wastewater, makes them cost-effective and sustainable agents for large-scale applications.

#### Microbial consortia–mediated bioremediation of azo dyes

The application of microbial consortia in wastewater treatment has been widely explored due to their superior degradation performance (Tables 2, 3 and 4). While individual microorganisms can degrade azo dyes, they rarely achieve full mineralization and often leave behind toxic aromatic amines as harmful intermediates. In contrast, microbial consortia exploit the synergistic and complementary metabolic pathways of multiple species, resulting in more efficient and resilient dye detoxification and mineralization, even under harsh environmental conditions [32]. Different microorganisms can act on distinct portions of the dye molecule or metabolize diverse intermediates generated during dye breakdown, creating a true synergistic effect where the enzymatic activity of one strain is enhanced by the presence of others, thereby markedly accelerating the overall degradation rate [33]. For instance, a study by Masarbo et al. [129] demonstrated that bacterial consortia composed of various combinations of *Bacillus* sp., *Lysinibacillus* sp., *and Kerstersia* sp. achieved superior decolorization efficiency and faster rates compared to the performance of the individual strains tested separately. Another study by Tian et al. [196] revealed that a halophilic bacterial consortium isolated from textile wastewater could achieve up to 98.2% degradation efficiency of the azo dye Metanil Yellow G, even under high-salinity conditions, highlighting its remarkable adaptability to extreme environments.

Not only bacterial consortia exhibit such phenomena, but other microbial groups also display remarkable synergistic capabilities in bioremediation. For instance, a study by El-Rahim et al. [156] demonstrated that a consortium of four fungal species—*Aspergillus niger*,* Aspergillus terreus*,* Aspergillus oryzae*,* and Aspergillus fumigatus*—achieved nearly complete decolorization and removal of two azo dyes, Direct Violet and Methyl Red. Moreover, cross-species collaborations can also enhance bioremediation efficiency. For example, *Aspergillus tamarii* and *Pseudomonas putida* (a fungal–bacterial consortium) [197], as well as *Saccharomyces cerevisiae* and *Fimbristylis dichotoma* (a yeast–plant consortium) [154], have been shown to efficiently degrade and decolorize azo dyes, reaching more than 90% of degradation in some instances. Algal and cyanobacterial bioremediation of azo dyes has likewise demonstrated this phenomenon, whether through pure algal or cyanobacterial consortia or through cross-species associations with each other or with other microorganisms. For example, *Chlorococcum* sp. and *Scenedesmus obliquus* (an algal consortium) [194], *Scenedesmus obliquus* and *Oscillatoria* sp. (an algal–cyanobacterial consortium) [183], and *Pseudomonas putida*, *Chlorella* sp., and *Lactobacillus plantarum* (an algal–bacterial consortium) [192] have each exhibited remarkable efficiency in bioremediation of various azo dyes, achieving decolorization and degradation rates exceeding 90%.

Interestingly, recent research highlights the pivotal role of co-metabolism in azo dye biodegradation and its intrinsic connection to microbial consortia. For example, An et al. [146] demonstrated that Direct Black G was degraded in a thermophilic microbial consortium via a co-metabolic pathway, in which reducing equivalents (e.g., NADH, FADH₂) generated through the tricarboxylic acid (TCA) cycle and glycolysis provided an extracellular reducing force that drove the dye’s breakdown. This study highlighted how microbial consortia, with their diverse and complementary metabolic networks, excel at co-metabolic processes: one species can supply reducing equivalents or essential intermediates that drive the dye-degrading activity of others, enabling faster, more efficient, and more complete detoxification of complex azo dyes, making consortia-based systems highly effective and sustainable for large-scale bioremediation applications [193, 198].

## Recent methods for azo dye biodegradation

Recent research on azo dye removal has increasingly shifted toward more advanced, integrated, and sustainable treatment approaches. These strategies aim to enhance degradation efficiency, target toxic intermediates, and achieve more complete detoxification of azo dyes in a cost-effective and environmentally sustainable manner.

### Genetically engineered microorganisms

Genetically modified microorganisms (GEMs) have been employed in azo dye biodegradation to enhance the efficiency of specific enzymatic steps within microbial degradation pathways. Using molecular techniques such as transposon mutagenesis, CRISPR–Cas9 genome editing, and recombinant DNA technology, GEMs can be engineered to express key enzymes such as azoreductase, laccase, and other oxidoreductases that facilitate reductive cleavage of azo bonds, ultimately leading to dye degradation and mineralization [199]. For example, genetically engineered *E. coli* expressing azoreductase from *Enterococcus faecalis* showed enhanced degradation rates for dyes such as Methyl Red and Reactive Black 5 under anaerobic conditions [200]. Another study explained that the transferred azoreductase enzyme-coding gene azoK from *Klebsiella pneumonia* to *Escherichia coli* achieved decolorization rate up to 95% for different azo dyes in less than 24 h [201]. GEMs may be modified to degrade not only the parent azo colouring molecule but also its toxic aromatic by-products, thereby reducing the persistence of toxic intermediates associated with mutagenicity and carcinogenicity. GEMs act in a sequential manner in one degradation pathway, with the first cleavage of the azo bond followed by oxidative metabolism to avoid the accumulation of toxic intermediates [199].

### Microbial fuel cells

Microbial fuel cells (MFCs) are bio-electrochemical systems that use microbial metabolism to oxidise organic pollutants and produce electricity as a by-product. In the context of the degradation of azo-dyes, MFCs are oxidative systems in which organic compounds are oxidized on the anode and the resulting electrons are transferred to the cathode, where they are reduced to oxygen as the final electron acceptor, and their reduction leads to bond cleavage and eventual degradation [202, 203]. Recent studies have demonstrated the high dyeing efficiency of modified MFC systems, enhanced by additive formulations. For example, MFC modules have demonstrated dual functionality as a platform for both decolourization and bioelectricity generation, achieving degradation efficiencies of up to 96.6% in 8 h for Allura Red [204]. In another study, a built-in wetland microbial fuel cell (CW-MFC) system achieved the highest decolourization efficiency, 96% for acid Red 18, outperforming conventional MFCs and bioreactors, especially in closed circuit configurations [28].

### Immobilization

Immobilization itself is a long-established concept in biochemical and environmental fields, but its inclusion among recent methods stems from advances in material science and bioreactor design. This approach relies on physically confining microbial cells or enzymes within or onto a solid support, such as alginate beads, biochar matrices, magnetic hydrogels, or nanocomposite polymers, without loss of catalytic activity [205, 206]. Recent advancements have significantly enhanced the stability and performance of immobilized biocatalysts. Actually, the main limitation of immobilized systems at larger scales is not reaction efficiency but biomass recovery. Conventional immobilization supports typically depend on filtration or centrifugation, which increases handling time and leads to progressive biomass loss. For this reason, magnetic nanoparticles based on iron oxide (Fe₃O₄) have attracted attention as functional supports, as they enable rapid and non-invasive separation of immobilized biomass using an external magnetic field. This operational advantage of magnetic separation has been widely recognized as a key factor facilitating biomass recovery and reuse in environmental bioprocesses [207]. In this context, a study by [208] showed that *Bacillus subtilis* immobilized on magnetite Fe₃O₄ nanoparticles achieved up to 80% degradation efficiency of Congo Red dye while maintaining its activity over seven reuse cycles.

Other immobilization strategies primarily target physical retention and structural stability. Alginate-based systems are widely used for this purpose. For example, *Scenedesmus quadricauda* achieved 100% decolorization of Indigo Blue dye after immobilization on sodium alginate [209]. Additionally, the microbial laccase/ 2,2’-azino-bis-(3-ethylbenzothiazoline)-6-sulfonic acid (ABTS) system, immobilized in layered double hydroxide/alginate biohybrid beads, achieved 92% degradation of the green colour of the dye within 120 min, with consistent activity over multiple reuse cycles [210]. Similarly, *Aspergillus terreus* immobilized in sodium alginate composite beads (containing 20% fungal biomass) reached a maximum adsorption capacity of 188 mg/g for Alizarin Red S dye under optimal conditions [211]. Novel polymers have also emerged as promising smart carriers for microbial immobilization. For instance, cells of *Ralstonia pickettii* immobilized in polyvinyl alcohol-alginate-hectorite beads, showed efficient adsorption and biodegradation of the methyl orange dye [212]. Furthermore, some supports combine microbial immobilization with additional adsorption capacity. Biochar matrices derived from agricultural residues fall into this category. A previous study reported that white rot fungi immobilized on corn straw biochar removed 96.17% of Acid Red G dye, benefiting from the porous structure of the biochar that enhanced fungal attachment and dye adsorption [213].

### Bioreactors

Recent research has shown that various bioreactor strategies can treat industrial wastewater containing dye. For example, a consortium of alkaliphilic microbial cultures from soda lakes, working in a two-stage continuous anaerobic and aerobic reactor, achieved a dye removal of about 99% of the synthetic textile effluent (and 96–98% for real effluent) with 87–93% COD and 90–96% removal of total Kjeldahl nitrogen (TKN) was observed ([177] b). Similarly, a thermophilic anaerobic stirred-tank reactor inoculated with digested sludge decolorized high-strength azo-dye solutions up to 90% in 7 days at 35 °C [214]. White-rot fungi immobilized in bioreactors showed similar results: in a continuously aerated reactor, the mycelium of *Pleurotus ostreatus* removed > 90% of the three-dye mixture after each cycle without increasing the toxicity of the waste stream [215]. Complementing these approaches, membrane bioreactors of all types have demonstrated high efficiency in removing azo dyes. By enabling cell or immobilization enzyme retention, bioreactor configurations increase biomass density and contact time, thereby protecting fungi and bacteria from hydraulic washout and environmental shocks, and sustaining long-term activity [216]. Both anaerobic membrane bioreactors (AnMBR) and submerged anaerobic membrane bioreactors (SAMBR) have shown high decolorization efficiency when applied to fungal or bacterial cultures. For example, an improved AnMBR system achieved 99% removal of Navy-Blue dye [217]. Another comparative study showed 61% removal of Reactive Orange 16, when compared to anaerobic biodegradation and ozonation, revealing that ozonation achieved higher decolorization, while combined treatments improved overall degradation efficiency [218]. A recent study demonstrated that the CSCM bioreactor achieved high azo dye decolorization efficiencies up to 98% for Acid Orange 7, 82% for Reactive Black 5, and 72% for Direct Blue 71 [219].

### Bionanotechnology and nanocatalysts

Recent studies have increasingly focused on nanotechnology for the remediation of azo dyes. Bio-based nanoparticles derived from plant extracts or microbes offer environmentally friendly synthesis pathways in addition to high surface activity and reactivity [220, 221]. The most recent studies for the removal of azo dyes and their derivatives using bio-nanoparticles are summarized in Table 5. Integrating nanotechnology with biodegradation addresses the key constraint of free-cell systems. These constraints include the difficulty of separating biomass from treated water and the risk of microbial secondary contamination [222]. In this context, magnetic nanomaterials are mainly used for process stability, recovery, and reuse rather than for biodegradation.

**Table 5:** Comparison of recent studies on adsorption and degradation of azo dyes by bio-nanoparticles

Nanoparticle type	Biological Source	Target azo dye(s)	Removal efficiency	Incubation time	Experimental conditions	References
AgNPs	*Bacillus pumilus*	Congo Red	96.99%	72 h	30 °C, static, 72 h; 10 ppm dye; 20 ppm Ag	Modi et al. [223]
	*Cestrum nocturnum* L.		> 80%	15 min	Reaction mixture: 4 mL of 1 mg mL⁻¹ dye + 200 µL of 0.5 M NaBH₄ + 200 µL AgNPs	Kumar et al. [224]
		Methylene Blue	79%	8 min		
AuNPs	*Eucommia ulmoides* bark extract	Methyl Orange	99.7%	3 min	50 °C, pH 9.0, ≤ 16 min; 0.5 mM HAuCl₄ + excess NaBH₄	Wan et al. [225]
		Reactive Orange 5	98.9%	6 min		
		Reactive Yellow 145	98.7%	6 min		
		Reactive Red 195	96.3%	6 min		
		Reactive Orange 1	99.4%	14 min		
		Reactive Yellow 179	98.6%	16 min		
		Acid Orange 7	91.1%	9 min		
Fe₃O₄NPs	*Piper chaba* stem extract	Congo Red	90%	125 min	pH 4.0, 20 mg/L dye, 20 mg IONPs, 0.1 L, 200 rpm	Yesmin et al. [226]
	Chitosan (crustacean biopolymer)	Methyl Orange	93.2%	40 min	25 °C; C₀ = 100 mg L⁻¹; pH range 4–10 (max at pH 8.0	Freire et al. [227]
		Reactive Black 5	99.9%		25 °C; C₀ = 100 mg L⁻¹; pH 4.0	
NiONPs	*Shewanella* spp. SM33	Methylene Blue	93.57%	4 h	Sunlight exposure; 1 mg mL⁻¹ NiO-NPs; static, shaking conditions	Mustafa et al. [95]
		Malachite Green	91.05%			
		Reactive Black 5	55.17%			
		Reactive Red-II	55.45%			
		Direct Blue-I	59.94%			
CuNPs	*Bacillus flexus* strain FB-1	Reactive Black-5	96.8%	4 h	Solar irradiation; 0.5 mg mL⁻¹ Cu-NPs; 25 ppm dye	Batool et al. [228]
		Congo Red	92.2%			
		Malachite Green	96.9%			
		Methylene Blue	72.5%			
		Reactive Red-2	58.5%			
		Blue Direct	78.7%			
ZnONPs	*Senna siamea* leaf extract	Reactive Red 141	100%	40 min	Contaminant concentration: 10 mg/L; Catalyst loading: 50 mg; Light source: Solar light	Thangsan et al. [229]
MgONPs	*Azadirachta indica* (Neem leaf extract)	Reactive Red 195	91%	90 min	pH 4; 40 °C; 0.02% dye concentration; 0.003 g/L catalyst	Kiran et al. [230]
MgONPs	*Aspergillus japonicus*	Reactive Black 5	90.4%	40 min	5 mg biosorbent; 100 mg L⁻¹ dye; pH 6; 25 °C; 150 rpm	El-Sharkawy et al. [231]

#### Magnetic nanoparticles in azo dye remediation

Magnetic nanoparticles, particularly iron oxide (Fe₃O₄), have been applied in azo dye remediation as multifunctional materials that integrate adsorption, catalytic activity, and magnetic recovery within a single system. Their practical value lies primarily in their superparamagnetic behavior and high surface area, which enable interaction with dye molecules and rapid separation from treated media using an external magnetic field [232]. This combination reduces reliance on conventional solid–liquid separation steps and improves operational handling. Fe₃O₄ nanoparticles are commonly synthesized through co-precipitation of Fe²⁺/Fe³⁺ salts, as well as hydrothermal or sol–gel methods, with increasing interest in green synthesis routes based on plant extracts [222]. These preparation approaches aim to maintain surface reactivity while preserving magnetic responsiveness, both of which are essential for repeated application in dye removal processes. After treatment, the particles can be magnetically recovered and reused with minimal structural disruption. For example, a green-synthesized Fe₃O₄ adsorbent retained approximately 89% of its dye removal capacity after four reuse cycles, corresponding to a loss of about 11%. This performance indicates that the contribution of Fe₃O₄ nanoparticles in azo dye remediation is predominantly operational, improving recovery, reuse, and process stability rather than introducing new degradation pathways [233]. As a result, Fe₃O₄-based nanomaterials are best viewed as robust and reusable supports for sequential azo dye treatment systems.

#### Advanced photocatalysts

Advanced photocatalysts are mainly introduced to address two persistent limitations of conventional TiO₂-based systems, namely restricted visible-light utilization and rapid electron–hole recombination. Therefore, current developments focus on modifying charge-generation and transfer processes rather than altering the fundamental chemistry of azo dye degradation [234, 235]. One common approach is doping TiO₂ or related oxides with metal or non-metal elements. This modification narrows the band gap and allows the catalyst to respond to visible light. For example, Mo-doped TiO₂ prepared with urea showed 99.2% degradation of Acid Black 1 within 120 min, while the undoped material reached only 73.7% under the same conditions [236]. Another strategy relies on heterojunction photocatalysts, where two semiconductors are combined to control charge movement. In ZnO/g-C₃N₄ S-scheme composites containing 10 wt% g-C₃N₄, about 95% degradation of a test pollutant was achieved within 1 h under visible light, which was attributed to improved charge separation [234]. These examples show that advanced photocatalysts mainly work by reducing energy losses during light-driven reactions. They increase reaction rates by improving photon use and limiting recombination.

#### Binary graphene and zeolite-based nanocomposites

Graphene or graphene oxide (GO)–zeolite nanocomposites have been investigated mainly as high-capacity adsorptive materials for azo dye removal, exploiting the complementary properties of both components. In these systems, graphene provides a π-conjugated surface that favors interaction with aromatic dye molecules, while zeolites supply a porous aluminosilicate framework with ion-exchange sites that support mass transfer and adsorption [237, 238]. Their combined action increases the uptake of colouring matter compared to the single component materials, and adsorption remains the predominant removal mechanism. Previous reports showed high removal efficiencies and good operational stability for such composites. For example, a magnetic GO–zeolite adsorbent removed 98.5% of methylene blue (MB, a model dye) from water within 20 min at near-neutral pH (fitting a Langmuir isotherm with R²≈0.996) [239]. In another case, a Cu–graphene-oxide/zeolite composite reached MB adsorption capacities up to ~ 97 mg/g (at 298 K, pH > 9) with pseudo-second-order kinetics and a Freundlich isotherm [240]. These improvements are generally attributed to the increased surface area and availability of active sites introduced by graphene rather than to fundamental changes in dye degradation pathways. Consequently, graphene–zeolite nanocomposites are best viewed as supporting materials that concentrate azo dyes and improve process efficiency, sometimes assisting downstream photocatalytic or redox treatments when additional active phases are present.

### Artificial intelligence and data-driven optimization

Artificial intelligence (AI) and machine learning (ML) have recently emerged as transformative tools in optimizing azo dye remediation processes. By integrating predictive modeling with experimental data, AI-based frameworks can predict dye degradation rates, identify influential operational parameters, and recommend optimal treatment conditions for different biological and hybrid systems [241, 242]. Algorithms such as Artificial Neural Networks (ANNs), Support Vector Machines (SVMs), Decision Trees (DTs), and Genetic Algorithms (GAs) are widely applied to simulate and optimize complex, nonlinear relationships governing dye degradation kinetics. For example, recent research by Ganthavee et al. [242] demonstrated that an ANN–SVM–Random Forest hybrid model accurately predicted the decolorization efficiency of a three-dimensional electrochemical reactor treating Methyl Orange, achieving approximately 98% removal under optimized conditions (115 mA cm⁻², 30 min, 50 mg L⁻¹). Similarly, Ahmad et al. [241] integrated Gene Expression Programming (GEP), Support Vector Regression (SVR), and Random Forest (RF) with a fruit-fly optimization algorithm to model the simultaneous biodegradation of azo dyes and hexavalent chromium by *Klebsiella* sp., demonstrating superior prediction reliability for coupled redox processes. AI tools have also been applied to photocatalytic and adsorption-based systems. Kohl et al. [243] used Bayesian optimization to fine-tune the temperature and flow parameters of a TiO₂-coated catalytic static mixer, achieving high-efficiency degradation of Reactive Orange 16 within only 21 h of automated operation. Collectively, these studies highlight how AI-driven modeling is transforming azo dye treatment from empirical optimization toward intelligent process control. When AI approaches are integrated with experimental workflows, azo dye remediation processes can become significantly more efficient and sustainable. In multiple cases, near-complete dye removal (≈ 100%) was accomplished under optimized, model-guided conditions [8].

### Integrated and hybrid Systems

Integrated hybrid and treatment systems combine complementary technologies to overcome the limitations of individual methods, achieving enhanced removal efficiency, more complete mineralization, and potential energy recovery [244, 245]. The combination of advanced oxidation processes (AOPs) and biological treatment can lead to near-complete decolorisation and total organic carbon (TOC) removal; UV/O₃ pretreatment of the reactive azo dye RR-198 achieved very high decolorization and TOC removal when followed by treatment in a sequencing-batch bioreactor [244]. Similarly, a combined Fenton–ozone (O₃/Fe²⁺/H₂O₂) process applied to real textile effluent resulted in approximately 89% color removal and 82% COD removal [245]. Photocatalytic membrane bioreactors (PMBRs), which integrate photocatalysis with membrane biofiltration, demonstrated enhanced performance in a pilot-scale study using tungsten-oxide/graphene-oxide beads. While photocatalysis alone removed 25% of color and 48% of COD, overall removal efficiencies increased to about 70% for color and 76% for COD after subsequent membrane biotreatment under optimized conditions [246]. Hybrid constructed wetlands (CW) coupled with microbial fuel cells (CW–MFCs) have shown promising results for azo dye treatment. In one study, a CW–MFC treating 100 mg/L of Reactive Red X-3B azo dye achieved approximatly 85.0% decolorization and 60% COD removal. Improvements in the removal efficiency of the examined azo dye were achieved by changing the external resistance. Under 100 Ω of external resistance and 800 mg/L of X-3B, the COD removal rate reached up to 78.0%, and the removal rate of the dye reached 85.2%. At this point, the system also generated electricity (peak powerdensity 0.024 W/m³) and naturally regulated pH, demonstrating the synergy of electrochemical and ecological processes [247].

## Future scope and challenges

Although biodegradation of industrial synthetic azo dyes has been recognized as a promising strategy, further investigation of its limitations is required to enhance efficiency for practical applications. Future studies should therefore focus on overcoming current limitations in bioremediation, optimizing bioreactor operating conditions, and achieving complete mineralization of azo dyes using sustainable practices. Most synthetic azo dyes remain difficult to fully mineralize because of their complex chemical structures. Appropriate biotechnological strategies should be developed and validated to ensure that azo dye molecules are degraded into environmentally safe by-products, and innovative molecular biology techniques should be used to detect the genes and enzymes responsible for the dye degradation process to provide researchers with deeper insights into bioremediation mechanisms. Also, currently used approaches and recent successful techniques should be re-examined to improve their bioremediation efficiency.

Biodegradation pathways and environmental conditions are critical factors influencing the removal of azo dye contaminants and should be carefully considered when designing effective degradation strategies. It is vital to reduce the possible toxicity of resulting metabolites, and if these metabolites can be recovered and used in other fields, research should focus on them. Modern computational techniques like quantum mechanics/molecular mechanics simulations and molecular docking may be applied to predict enzyme–dye interactions. Moreover, it is crucial to explore novel uses for azo dyes removal from both synthetic and real industrial effluents at a commercial scale. Finally, advancing novel approaches across different treatment strategies is essential to leverage the unique strengths of each method and integrate them into more effective hybrid systems, enabling industries to adopt scalable solutions for azo dye degradation and sustainable wastewater management.

## Conclusion

Microbial bioremediation represents a powerful and sustainable strategy for mitigating the environmental and health risks associated with azo dye pollution. Through mechanisms such as enzymatic catalysis, biosorption, bioaccumulation, and co-metabolic interactions, diverse microorganisms are capable of transforming complex dye molecules into less harmful end products. The effectiveness of these microbial systems is further enhanced by the synergistic activity of microbial consortia, which combine complementary metabolic pathways to achieve more efficient and complete dye degradation. Recent advances in biotechnology—including genetic engineering, immobilization technologies, advanced bioreactors, nanotechnology, and artificial intelligence-assisted process optimization—have significantly improved the stability, efficiency, and scalability of microbial azo dye remediation processes. Despite these advances, several challenges remain, including the formation of toxic degradation intermediates, reduced microbial performance under harsh industrial wastewater conditions, and the need for improved understanding of microbial metabolic pathways involved in dye degradation. Future research should therefore focus on developing robust microbial systems and engineered consortia capable of operating under complex environmental conditions while ensuring complete detoxification of dye contaminants. Overall, integrating mechanistic insights with emerging biotechnological innovations will accelerate the development of efficient microbial systems for sustainable azo dye remediation and environmentally responsible wastewater management.

## Data Availability

Data will be given upon request.
